# Comparison of cleaning Efficacy and Instrumentation Time between Rotary and Manual Instrumentation Techniques in Primary Teeth: An *in vitro* Study

**DOI:** 10.5005/jp-journals-10005-1347

**Published:** 2016-06-15

**Authors:** Farhin Katge, Vamsi Krishna Chimata, Manohar Poojari, Shilpa Shetty, Bhavesh Rusawat

**Affiliations:** 1 Professor and Head, Department of Pedodontics and Preventive Dentistry, Terna Dental College, Navi Mumbai, Maharashtra, India; 2 Lecturer, Department of Pedodontics and Preventive Dentistry, Terna Dental College, Navi Mumbai, Maharashtra, India; 3 Reader, Department of Pedodontics and Preventive Dentistry, Terna Dental College, Navi Mumbai, Maharashtra, India; 4 Lecturer, Department of Pedodontics and Preventive Dentistry, Terna Dental College, Navi Mumbai, Maharashtra, India; 5 Lecturer, Department of Pedodontics and Preventive Dentistry, Terna Dental College, Navi Mumbai, Maharashtra, India

**Keywords:** Hedstrom files, Primary teeth, Rotary instrumentation.

## Abstract

**Objectives:** The aim of this study was to compare the cleaning efficacy and instrumentation time between manual Hedstrom files (H-files) and rotary Mtwo files in primary molar root canals.

**Materials and methods:** A total of 90 primary root canals were selected using standardized radiographs. The canals were injected with India ink with 30 gauge insulin syringe and divided into three groups. Group I―30 root canals instrumented with H-files, group II―30 root canals instrumented with Mtwo files, and group III―control group in which no canal instrumentation was done. The teeth were cleared in various solutions and then observed under a stereomicroscope.

**Results:** No significant difference was seen in cleaning efficacy between H-files and Mtwo files in coronal, middle, and apical thirds of the root canal. The instrumentation time recorded for H-files (3.41 ± 0.38 minutes) was significantly less than that of Mtwo files (4.81 ± 0.52).

**Conclusion:** Although there was no significant difference in cleaning capacity, further studies should be carried out using the single file systems.

**How to cite this article:** Katge F, Chimata VK, Poojari M, Shetty S, Rusawat B. Comparison of cleaning Efficacy and Instrumentation Time between Rotary and Manual Instrumentation Techniques in Primary Teeth: An *in vitro* Study. Int J Clin Pediatr Dent 2016;9(2):124-127.

## INTRODUCTION

The premature loss of primary teeth may cause changes in the chronology and sequence of eruption of permanent teeth. Maintenance of primary teeth until physiological exfoliation contributes to mastication, phonation, and esthetics and prevents deleterious habits in children.^1^

Therefore, primary teeth with pulpitis or necrosis are indicated for endodontic treatment.^2^

The success of endodontic therapy is directly related to the microbial reduction in the root canal system through root canal debridement, shaping, and sealing.^3^ Manual instrumentation for cleaning root canals can be done by K-files and Hedstrom files (H-files). Hedstrom files are recommended since they remove hard tissue only on withdrawal and penetrate readily with a minimum of resistance, which prevents pushing infected material through the apices.^4,5^

Recently, endodontics has been revolutionized with the introduction of rotary nickel-titanium (NiTi) systems. The use of rotary instrumentation in permanent teeth has proven to be efficient with decreased instrumentation time in atretic and curved molar root canals.^6,7^ They not only provide greater flexibility, but also raise the possibility of automated instrumentation.^8^

Thus, the aim of this study was to compare the cleaning efficacy and instrumentation time between manual (H-files) and rotary (Mtwo) instrumentation techniques in primary teeth.

## MATERIALS AND METHODS

This study was approved by the Research Ethics Committee of the institution where the study was conducted. A total of 50 extracted primary molars with at least two-thirds of the root intact were washed in water and stored in 3% sodium hypochlorite solution for 1 week for disinfection. The reasons for extraction were infected primary molars with significant amount of bone loss, root resorption of one of the roots with the other roots intact because of altered path of eruption of the permanent successor, and over-retained primary molars.

### Sample Selection

A total of 90 mesial and distal canals without external or internal resorption and canal calcification were selected for the study. Coronal access was achieved using a large round diamond bur (BR-46; Mani Inc., Japan) followed by irrigation of pulp chamber and root canals with 3% sodium hypochlorite solution. Digital radiographs were taken in lab with #10 K-file (Dentsply-Maillefer, Ballaigues, Switzerland) introduced into the root canal 1 mm short of the apex or the root bevel for working length determination and to check the patency of the root canals. India ink was injected with a 30-gauge insulin syringe into the root canals. A K-file size #10 was inserted into the canal to assure penetration of the dye through the canal and the teeth were then stored in wet conditions for 48 hours. The root canals were randomly divided into three groups:

*Group I (N=30):* The root canals were prepared manually using H-files (Mani Inc., Japan)

*Group II (N=30):* The root canals were instrumented with rotary Mtwo files (VDW, Munich, Germany)

*Group III (N=30):* Control group canals were not instrumented at all.

### Preparation of Canals

The canals were prepared by a single operator who was experienced in both manual and rotary instrumentation. Manual instrumentation with H-files was achieved by in and out filing motion. The preparation was completed using step-back technique with files of size 15 to 30 with recapitulation.

Rotary canal preparation was done with 21 mm length Mtwo NiTi files driven by an Endo-mate DT (NSK, Nakanishi, Japan) hand piece at speed between 250 and 350 rpm as recommended by the manufacturer. A total of four Mtwo instruments (10/0.04, 15/0.05, 20/0.06, and 25/0.06) were used to prepare canals up to the determined working length of the root canals of the molar teeth. Each instrument was used five times and then discarded. During instrumentation, the root canals were irrigated with 5 ml of 3% sodium hypochlorite solution.

After drying each canal with sterile paper points, the pulp chamber was sealed with temporary cement (Coltosol, Coltene Whaledent, Switzerland) and the apical end with sticky wax.

The instrumentation time in each canal was measured with a chronometer. Even the time taken for instrumentation exchange was considered.

### Scoring of the Canals

The teeth were decalcified by immersing them in 7% hydrochloric acid for 2 days. The acid solution was changed each day. After decalcification, the teeth were washed under running water. The teeth were then immersed in a series of diluted ethyl alcohols for dehydration: Initially in 70% alcohol for 16 hours (changed every 8 hours) followed by 80% alcohol for 8 hours, 95% alcohol for 8 hours, and 100% alcohol for 8 hours. The dehydrated teeth were then cleared by immersing them in methyl salicylate for 6 hours.

**Fig. 1:**
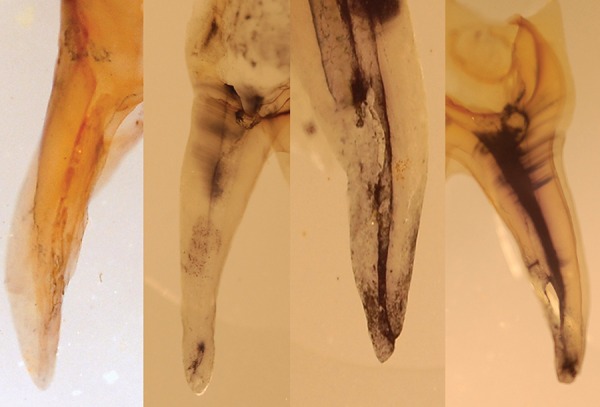
Grading 0, 1, 2, and 3 of canals after making the tooth transparent for analysis

The canals were analyzed by an observer who was unaware of the groups (blinded) under a stereomicroscope (SMZ-143 series, Motic Company) at 10x magnification for remaining traces of India ink in coronal, middle, and apical third of the canals. The scoring criteria used were as follows (Fig. 1):

*Score 0:* total clearing (canal was completely clean)

*Score 1:* almost complete ink removal (traces of ink in some areas)

*Score 2:* partial ink removal (remnants of ink found on some walls in some areas)

*Score 3:* no ink removal (appreciable amount of ink present)

Statistical analysis was done using MedCalc Statistical Software version 12.7.2 (MedCalc Software bvba, Ostend, Belgium; http://www.medcalc.org; 2013). The scores obtained were analyzed with Mann-Whitney “U” test. The significance of values for instrumentation time was done using Student’s independent t-test.

## RESULTS

**Graph 1:**
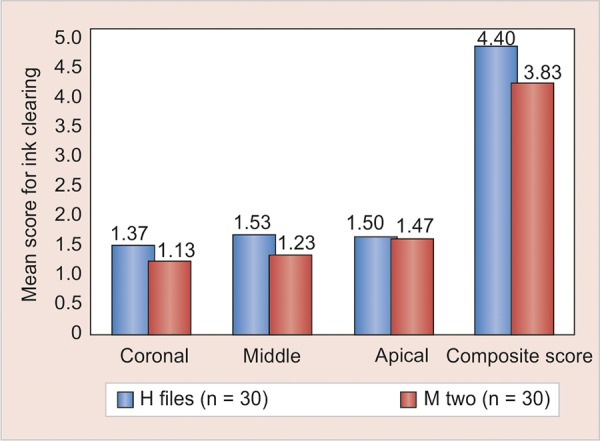
Mean score of ink clearing at coronal, middle, and apical third of the canals and the composite score

On comparison between the control and experimental groups, it was proved that ink could not be removed without instrumentation. The mean scores of the remaining ink in the coronal, middle, and apical third of the canals are as shown in Graph 1.

In the coronal third of the root canals, H-files showed better cleaning efficacy than Mtwo files, but the difference was not statistically significant. The same results were seen even in the middle third, whereas in the apical third both the files showed the same cleaning efficacy.

The mean instrumentation time was shorter for H-files as compared with Mtwo files, and the difference was statistically significant (Table 1).

## DISCUSSION

Endodontic procedures for the treatment of primary teeth with necrotic pulps are indicated if the canals are accessible and if there is evidence of normal supporting bone.^4^ Rotary biomechanical preparation of deciduous teeth was first described by Barr et al^9^ who described the advantages and disadvantages of using rotary files in primary teeth. The authors considered this technique a more effective way to debride the uneven walls of primary teeth and to facilitate a consistently dense fill. Increased efficiency in both preparation time and root canal shaping helps maintain patient cooperation by diminishing fatigue, thus increasing clinical success.^10^

Previous studies have been carried out on the same topic but using K-files for manual instrumentation. In this study, H-files were chosen to study their effectiveness in cleaning efficacy as compared with Mtwo files, and there was no significant difference between the two instrumentation techniques, as also observed by Barr et al^9^ and Silva et al.^11^ The sequence of instrumentation for Mtwo files was as recommended by the authors.^12^

No instrumentation was done in the control group to assure the proper penetration of India ink into the canals.

The instrumentation time recorded for manual instrumentation using H-files was less than that of rotary instrumentation, that is, Mtwo files. This was in contradiction to some articles but in accordance with Madan et al,^13^ and this difference in instrumentation time between manual and rotary instrumentation was considered as a matter of operator’s experience. The reason for lesser instrumentation time in this study maybe because the time for exchange of instruments was also recorded. Moreover, the number of instruments used which were four in each canal was also responsible for the increased instrumentation time. Mtwo files were primarily designed for permanent molars and the need to use all the files in primary molars is skeptical and requires more insight.

**Table 1:** **Table 1:** Comparison of instrumentation time between H-files and Mtwo rotary files

		*Mean*		*SD*				*Mean* *difference*		*95% CI* *Lower*		*95% CI* *Upper*	
H-files		3.41		0.38		H-files *vs* Mtwo files		1.40		1.00		1.11	
Mtwo file		4.81		0.52									
p-value		< 0.0001											

SD: Standard deviation; CI: Confidence interval; Statistically significant difference according to t-test (p < 0.0001)

## CONCLUSION

Root canal treatment can be carried out in primary teeth using both rotary and manual instrumentation. According to the findings in the current study, both were equally effective in cleaning the primary root canals, and the instrumentation time recorded was less for H-files as compared with rotary instrumentation. Still, the author is of the view that further studies should be carried out using the newer single NiTi file systems where the instrumentation time maybe significantly reduced and hence advantageous to the clinician when treating children.
